# Validation of a Broad-Range Conventional RT-PCR Assay for Pestivirus Surveillance and Molecular Detection of BVDV-2a in Pigs in Costa Rica

**DOI:** 10.3390/v18070762

**Published:** 2026-07-11

**Authors:** Bernal León, Idania Chacón, Eunice Víquez, Olga Aguilar, Guisella Chaves, Tamara Solorzano, Carlos Jiménez, John Pasick, Llilianne Ganges

**Affiliations:** 1 LSE, Laboratorio Nacional de Servicios Veterinarios, Servicio Nacional de Salud Animal, Servicio Nacional de Salud Animal, Heredia 40104, Costa Rica; ichacon@senasa.go.cr (I.C.); eviquez@senasa.go.cr (E.V.); oaguilara@senasa.go.cr (O.A.); gchavesg@senasa.go.cr (G.C.); 2 Laboratory of Virology, Tropical Disease Research Program, School of Veterinary Medicine, Universidad Nacional, Heredia 40104, Costa Rica; tamara.solorzano.scott@una.cr (T.S.); carlos.jimenez.sanchez@una.cr (C.J.); 3 Formerly of the Canadian Food Inspection Agency, National Centre Foreign for Animal Disease, 1015 Arlington Street, Winnipeg, MB R3E 3M4, Canada; jmpasic55@gmail.com; 4 WOAH Reference Laboratory for Classical Swine Fever, Institute of Agrifood Research and Technology, Centre de Recerça en Sanitat Animal (CReSA), 08193 Bellaterra, Spain; llilianne.ganges@irta.cat; 5 IRTA, Programa de Sanitat Animal, Centre de Recerca en Sanitat Animal (CReSA), 08193 Bellaterra, Spain

**Keywords:** pestivirus PCR, diagnostic validation, in-house test, BVDV-2a, Costa Rica

## Abstract

The genus *Orthopestivirus*, within the family *Flaviviridae*, includes important pathogens such as classical swine fever virus (CSFV), bovine viral diarrhea virus types 1 and 2 (BVDV-1 and BVDV-2), and border disease virus (BDV). Although Costa Rica is endemic for BVDV, it remains officially free of CSFV and BDV, highlighting the need for reliable molecular tools for pestivirus surveillance and differential diagnosis in swine populations. This study validated a conventional broad-range in-house RT-PCR assay targeting the 5′ untranslated region (5′UTR) of pestiviruses, a genomic region that is highly conserved among members of the genus *Orthopestivirus*. A wide variety of PCR-based assays have been developed for pestivirus detection; however, many are designed specifically for the diagnosis of bovine pestiviruses, such as bovine viral diarrhea virus type 1 (BVDV-1), BVDV-2, and HoBi-like pestivirus (HoBiPeV) in cattle. In contrast, the assay described in this study was standardized and validated to detect multiple pestivirus species, thereby providing a useful tool for surveillance and diagnostic applications across different host species. A total of 66 reference samples were analyzed to determine diagnostic sensitivity and specificity, while analytical sensitivity, repeatability, reproducibility, and selectivity were also evaluated. Additionally, pestivirus surveillance was conducted from 2014 to 2025. The assay showed a limit of detection of 1.2 copies/µL for CSFV and 11.7 copies/µL for BVDV, with diagnostic sensitivity and specificity of 98% and 100%, respectively. A total of 1072 surveillance samples were tested, including 11 bovine and 1061 porcine samples. Two porcine samples collected in 2015 were positive for pestivirus and clustered within the BVDV-2a group, representing the first molecular detection of BVDV-2a in pigs in Costa Rica. Circulation of BVDV-1b in cattle was also confirmed. The assay demonstrated satisfactory performance as a broad-range pestivirus surveillance tool and may support differential diagnosis in CSFV-free countries when combined with sequencing confirmation for pestivirus species identification.

## 1. Introduction

The genus *Orthopestivirus*, within the family *Flaviviridae*, comprises several economically important RNA viruses that infect domestic and wild animals. Recent taxonomic revisions have led to the reclassification of pestiviruses into the family *Pestiviridae* and the genus *Orthopestivirus* [1]; however, the conventional nomenclature based on the genus *Pestivirus* is used throughout this manuscript for clarity and consistency with the diagnostic and veterinary literature. These viruses are responsible for substantial economic losses worldwide due to reproductive disorders, respiratory disease, immunosuppression, reduced productivity, and restrictions on international trade. Important members of the genus include classical swine fever virus—CSFV (*Orthopestivirus suis*), bovine viral diarrhea virus types 1—BVDV-1 (*Orthopestivirus bovis*) and 2—BVDV-2 (*Orthopestivirus tauri*), and border disease virus BDV (*Orthopestivirus ovis*), which affect swine, cattle, and small ruminants, respectively [2]. In cattle, BVDV infections range from subclinical presentations to severe systemic disease, including reproductive failure and the generation of persistently infected animals that play a central role in viral maintenance and transmission within herds [3,4,5]. Border disease virus mainly affects small ruminants and is associated with infertility, abortion, stillbirths, and congenital disease [6]. In swine, CSFV remains one of the most important transboundary animal diseases because of its high contagiousness, severe economic impact, and implications for international trade and food security [7]. Comprehensive reviews on CSFV pathogenesis, epidemiology, and control have highlighted the continuing importance of reliable molecular surveillance tools for disease prevention and eradication programs [8].

The clinical and molecular similarities among pestiviruses represent an important diagnostic challenge, particularly in surveillance systems aimed at the early detection of CSFV. In countries officially free of CSFV, differential diagnosis is essential because non-CSFV pestiviruses may occasionally infect pigs or be detected in porcine samples, potentially complicating surveillance and outbreak investigations. Costa Rica is endemic for BVDV but remains officially free of CSFV and BDV. Therefore, surveillance programs require diagnostic tools capable of detecting a broad range of pestiviruses while maintaining high diagnostic specificity in the context of CSFV surveillance.

Several highly sensitive real-time RT-PCR assays have been developed for the detection of CSFV and other pestiviruses, including broad-range assays targeting conserved regions of the 5′ untranslated region (5′UTR) [9,10]. These methods provide excellent analytical sensitivity and are widely used in reference laboratories. In addition, broad-range real-time RT-PCR systems and multiplex assays for pestivirus detection have been further optimized in recent years to support differential diagnosis and surveillance activities [10,11,12]. However, their implementation may be limited in some surveillance settings due to operational costs, equipment availability, and dependence on fluorescent probe-based platforms.

Conventional RT-PCR assays continue to represent a practical alternative for surveillance laboratories when they are properly validated, reproducible, and analytically robust. Their utility is particularly relevant in regional surveillance systems and in laboratories where broad-range detection and subsequent sequence characterization are required to differentiate CSFV from other pestiviruses detected in porcine samples. Previous studies have also demonstrated that viral detection during the early stages of CSFV infection may vary depending on the sample type, viral strain, and stage of infection, emphasizing the importance of reliable molecular tools for surveillance and differential diagnosis [8,13].

In the present study, we validated a broad-range conventional in-house RT-PCR assay targeting a conserved region of the pestivirus 5′UTR. The assay was evaluated using reference materials and interlaboratory proficiency testing samples representing CSFV, BVDV, and BDV, and its analytical sensitivity, diagnostic sensitivity and specificity, repeatability, reproducibility, and selectivity were assessed. Additionally, the assay was applied to pestivirus surveillance samples collected in Costa Rica between 2014 and 2025 to evaluate its practical utility under field diagnostic conditions and to investigate the occurrence of pestiviruses in porcine surveillance samples.

## 2. Materials and Methods

### 2.1. Samples and Reference Controls Used in This Study

In 2026, a total of nine bovine samples from five farms were analyzed by viral isolation in Madin–Darby Bovine Kidney (MDBK) cells. Seven samples were obtained from beef-production cattle and two from dairy-production cattle. A cytopathic effect (CPE) was observed in all samples, characterized by loss of cell adhesion and the presence of individual cells with a spindle-shaped, refractile morphology. The presence of bovine viral diarrhea virus was confirmed by direct immunofluorescence (DIF) using a fluorescein isothiocyanate conjugated polyclonal anti-BVDV antibody (provided by (code 243-FA; United States Department of Agriculture, NVSL, Ames Iowa, IA, USA)). Direct immunofluorescence was performed following the guidelines of the WOAH Terrestrial Manual for bovine viral diarrhea virus detection [14], with minor modifications, at Virology Laboratory of the School of Veterinary Medicine of the National University. This panel of samples was then subjected to RT-PCR assay at LANASEVE and confirmed by sequencing.

Reference samples (66) with known status for CSFV, BDV, or BVDV were extracted using the Dneasy blood and tissue kit protocol (Qiagen, Leipzig, Germany) and then analyzed. Of these, 42 samples were derived from interlaboratory proficiency tests conducted between 2015 and 2025; 15 were obtained from the Canadian Food Inspection Agency, National Centre for Foreign Animal Disease, in 2012; and nine samples, analyzed in 2026, were sourced from the sample bank of the Virology Laboratory of the School of Veterinary Medicine at the National University, and had been previously characterized during the standardization and initial validation of this RT-PCR assay. The list of reference controls used in this validation is provided in Supplementary Table S1.

### 2.2. Pestivirus in House RT-PCR

A total of 80 pestivirus sequences were retrieved from GenBank [15], including 8 BDV strains, 45 CSFV, 22 BVDV type 1 strains, and 5 BVDV type 2 strain sequences. These sequences were aligned to identify conserved regions for primer design using the BioEdit program [16]. The conventional in-house pestivirus RT-PCR assay used the forward primer 5′-ACGTGGACGAGGGCATG-3′ (positions 128–144) and reverse primer 5′-CAACTCCATGTGCCATGTACA-3′ (positions 266–286), which amplified a conserved fragment within the 5′ untranslated region (5′UTR) of viruses belonging to the genus pestivirus. The expected amplicon size is 159 bp for BVDV (PV233541, PX941914) and giraffe pestivirus (AF144617), 157 bp for CSFV (LC881260, LC881499) and reindeer pestivirus (AF144618), 158 bp for BDV (NC_003679), and 160 bp for *Pestivirus brasiliense*, according to reference sequence (OQ411021).

Amplification of the J BVDV and E CSFV positive controls (Table S1) was performed using the OneStep RT-PCR Kit (QIAGEN^®^, Leipzig, Germany). The final concentration of each primer was 0.3 µM, and 1 mM MgCl_2_ was added. A total of 4 µL of RNA per sample was used in a final reaction volume of 12.5 µL. The amplification protocol consisted of reverse transcription at 50 °C for 30 min, initial denaturation at 95 °C for 15 min, followed by 40 cycles of 94 °C for 10 s, 57 °C for 40 s, and 72 °C for 20 s, with a final extension at 72 °C for 5 min. The equipment used included two Veriti thermocyclers and two 2720 conventional thermocyclers manufactured by Applied Biosystems, Foster City, CA, USA.

### 2.3. Validation of the Pestivirus in House RT-PCR

#### 2.3.1. Limit of Detection (LOD) Copies/µL

After amplification of positive controls E and J, PCR products were analyzed by agarose gel electrophoresis, and the corresponding bands were excised. DNA was purified using the QIAquick Gel Extraction Kit (Qiagen, Leipzig, Germany), and concentrations of the eluted DNA were measured as 0.440 ng/µL for CSFV and 0.216 ng/µL for BVDV using a Quantus Fluorometer (Promega, Madison, WI, USA), with measurements performed in duplicate. Both controls were confirmed by sequencing Sanger method.

The number of DNA copies per microliter was estimated using a DNA copy number calculator https://www.technologynetworks.com/tn/tools/copynumbercalculator (accessed on 16 December 2025). Subsequently, ten-fold serial dilutions of both controls were prepared and analyzed using the pestivirus RT-PCR assay to determine the LOD.

#### 2.3.2. CSF Honduras Strain TCID_50_/mL

Ten-fold serial dilutions of the Honduras strain (titer: 10^5.50^ TCID_50_/mL) obtained from the serum bank of the National Centre for Foreign Animal Disease (NCFAD), were prepared in 10% homogenized tonsil tissue previously confirmed negative for the virus in the BSL3 laboratory at the NCFAD, Winnipeg, Canada. Viral RNA was extracted using the QIAamp Viral RNA Mini Kit according to the manufacturer’s instructions. In parallel, tenfold serial dilutions of the virus stock were prepared and processed using the in-house RT-PCR assay to compare the sensitivity and efficiency of the complete PCR protocol with those obtained from virus dilutions in homogenized tissue.

#### 2.3.3. RT-qPCR for CSFV RNA Detection

In cases of disagreement between the results of the reference laboratory and the conventional pestivirus RT-PCR assay, the Hoffmann RT-qPCR test was used as an additional comparative method to further evaluate samples with low viral loads. The assay was performed at LSE-LANASEVE using the OneStep RT-PCR Kit (QIAGEN^®^, Leipzig, Germany) and amplification conditions routinely implemented in the laboratory for CSFV molecular diagnosis, including QuantStudio 1 and a QuantStudio6 flex thermocycler models manufacturer by Applied Biosystem, Foster City, CA, USA. According to the laboratory diagnostic criteria, samples with Ct values below or equal to 35 are considered positive, while samples with Ct values higher than 35 were interpreted as inconclusive/suspect and were retested. This assay was selected because it is one of the RT-qPCR methods recommended by WOAH for the sensitive and specific detection of CSFV RNA [9].

PCR reactions were performed in a final volume of 16 µL using the OneStep RT-PCR Kit (QIAGEN^®^, Leipzig, Germany). Each primer was used at a final concentration of 0.7 µM, the CSF Hoffmann probe at 0.5 µM, and 0.4 mM MgCl_2_ was added. A total of 5 µL of RNA per sample was included in each reaction. The amplification protocol was 30 min at 50 °C and then 15 min at 95 °C, followed by 42 cycles of 15 s at 94 °C, 30 s at 57 °C, and 30 s at 68 °C.

Positive samples were confirmed by sequencing and analyzed using BLAST v2.17.0. Similar sequences were downloaded and aligned, and a phylogenetic tree was constructed using the K2 + I substitution model and the maximum likelihood method in MEGA12 with 1000 bootstrap replications [17].

#### 2.3.4. CSFV Reference Sample Panel for Diagnostic Sensitivity Evaluation

Diagnostic sensitivity was assessed using previously characterized CSFV-positive reference samples obtained from international reference laboratories, including the National Centre for Foreign Animal Disease (NCFAD, Winnipeg, MB, Canada), a WOAH Reference Laboratory for Classical Swine Fever, and IRTA-CReSA (Spain). The panel included archived blood samples collected at different days post-inoculation from pigs experimentally infected with the Diepholz, Honduras, and Peru strains, as well as additional reference materials previously characterized by the contributing laboratories. No experimental animal procedures were conducted for the purposes of the present study.

RNA was extracted using the QIAamp RNA Kit (Qiagen, Leipzig, Germany) according to the manufacturer’s instructions and analyzed by pestivirus RT-PCR to determine the earliest detection of classical swine fever virus in blood post-inoculation. The diagnostic sensitivity and specificity of the assay were determined using 66 reference control samples, as described in Table S1.

#### 2.3.5. Repeatability

Two replicates of reference samples M2 CSFV Kanagawa strain and M13 ASFV Lillie strain (NCFAD, Winnipeg, MB, Canada) and J DVB NADL (IRTA-CRESA Lab Catalunya, Cerdanyola del Vallès, Spain) were amplified under identical conditions on the same day.

#### 2.3.6. Reproducibility

Two replicates of reference samples M2 CSFV Kanagawa strain and M13 ASFV Lillie strain (NCFAD, Winnipeg, MB, Canada) and J DVB NADL (IRTA-CRESA Lab Catalunya, Spain) under varying conditions, including different operators, equipment (Veriti and 2720 thermocycler models), and days were tested by the RT-PCR.

#### 2.3.7. Selectivity

Selectivity was evaluated using replicate samples spiked with target and non-target nucleic acids. Two replicate samples were spiked with African swine fever virus and Dengue virus, respectively, as non-target controls. Additionally, replicate negative tonsil tissue samples were spiked with target nucleic acid and with non-target nucleic acid to assess potential cross-reactivity and matrix interference.

#### 2.3.8. Pestivirus RT-PCR Surveillance

Furthermore, from 1 January 2014 to 31 July 2024, a total of 579 samples were submitted to the Laboratorio LSE de Salud Animal, LANASEVE and analyzed using the in-house pestivirus RT-PCR assay. These included 568 tissue samples collected from pigs (tonsils and lymph nodes) and 11 blood samples obtained from cattle.

Additionally, within the framework of the PROCINORTE project, 272 samples originating from 247 farms were collected between August and December 2024, while 221 samples from 175 farms were collected between January and 9 September 2025. In total, 493 tissue samples were obtained from slaughterhouses. These samples included tissues from clinically healthy pigs as well as animals that died from unknown causes during transport or while held in lairage pens prior to slaughter. Many of these deaths were associated with stress-related conditions during handling and transportation. A smaller proportion of samples was collected from pigs condemned at slaughter due to lymphadenitis, cachexia, hemorrhagic lesions, erysipelas, and other pathological conditions. These samples were incorporated into active surveillance activities conducted under the PROCINORTE initiative.

All samples were processed using the DNeasy Blood and Tissue Kit (Qiagen, Leipzig, Germany) following the manufacturer’s instructions, and subsequently analyzed using the pestivirus RT-PCR assay.

## 3. Results

### 3.1. Limit of Detection (LOD) Copies/µL

The limit of detection (LOD) of the in-house RT-PCR assay targeting pestivirus was determined using two methods: a ten-fold serial dilutions of quantified positive controls expressed in copies/µL, and as 50% tissue culture infectious dose per milliliter (TCID_50_/mL). The initial concentrations for the first method were established at 2.55 × 10^9^ copies/µL for classical swine fever virus and 1.25 × 10^9^ copies/µL for bovine viral diarrhea virus.

**Figure 1:**
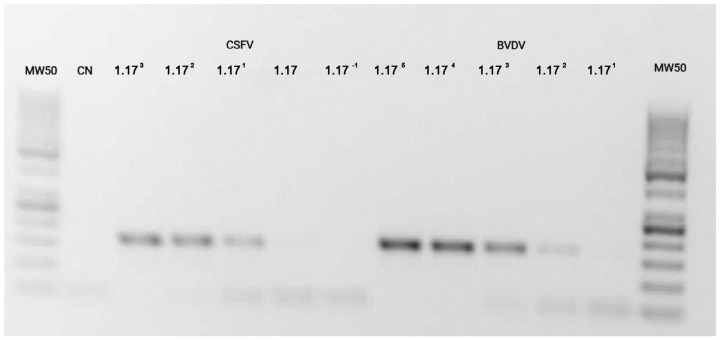
LODs for CSFV and BVDV; positive amplicons are observed between 150 and 200 bp. CN indicates the negative control. The first five lanes correspond to the initial serial dilutions of the CSFV strain, while the last five lanes correspond to the BVDV dilutions. A 50 bp DNA ladder was used as a molecular size marker, with the first prominent band corresponding to 250 bp.

The LOD was defined as the lowest dilution at which a visible amplicon of the expected size was detected. For CSFV, amplification was observed up to the [10 + 1] dilution, corresponding to approximately 1.2 copies/µL. For BVDV, amplification was detected up to the [10 + 2] dilution, corresponding to approximately 11.7 copies/µL. No amplification was observed at higher dilutions; Figure 1 illustrates the LOD for both CSFV and BVDV across the serial dilutions.

### 3.2. CSF Honduras Strain TCID_50_/mL

**Figure 2:**
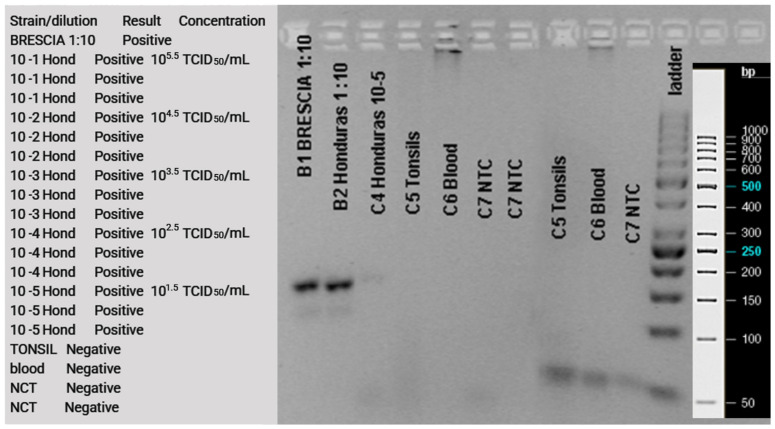
Serial dilutions of a Pestivirus Honduras strain expressed in TCID_50_/mL are shown in the right side of the figure. The lowest dilution detected by RT-PCR was 31.6 TCID_50_/mL, corresponding to a faint band. Lanes C5 and C6 represent negative controls (tonsil and blood matrices) run in duplicate, while lane C7 corresponds to a reactive control (water).

The second method was applied only to the CSFV Honduras strain. The initial dilution (10^−1^; 10^5.5^ TCID_50_/mL) corresponded to 316,227 TCID_50_/mL, while the last detected dilution (10^−5^; 10^1.5^ TCID_50_/mL) corresponded to 31.6 TCID_50_/mL. This is equivalent to 0.12 TCID_50_ per PCR reaction (Figure 2).

**Figure 3:**
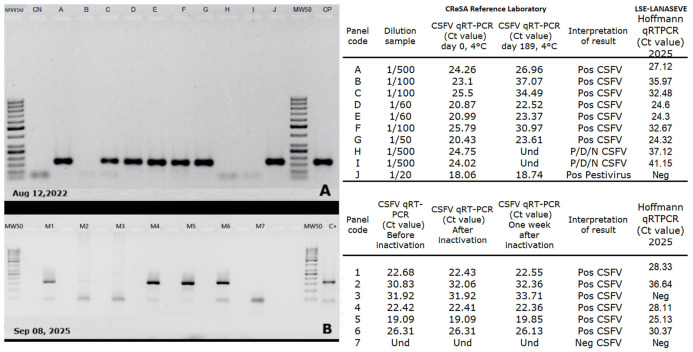
Interlaboratory comparison results for samples not detected by the pestivirus RT-PCR assay. In the PCR results, “Und” means undetermined, “Pos” or “P” positive, “Neg” or “N” negative, and “D” doubtful. (**A**) On the left, the CReSA reference panel analyzed at the LSE Virology Laboratory in 2022 using the conventional pan-pestivirus RT-PCR assay is shown. On the right, the third column presents the Ct values obtained at the CReSA laboratory on Day 0, when the reference panel was prepared. The fourth column shows the Ct values obtained after 189 days of refrigerated storage using the Hoffmann RT-qPCR assay. The final column presents the Ct values obtained when the same samples were reanalyzed in 2025 at the LSE Virology Laboratory using the same Hoffmann RT-qPCR assay. (**B**) On the left, the results of the conventional pan-pestivirus RT-PCR assay obtained with the 2025 CReSA reference panel are shown. On the right, the Ct values obtained at three different time points before and after sample inactivation are presented. The final column shows the Ct values obtained when the same samples were analyzed at the LSE Virology Laboratory in 2025 using the Hoffmann RT-qPCR assay.

Only four out the 50 positive samples were not detected by the pestivirus RT-PCR, Figure 3A,B on the left, show the agarose gel results of the IRTA-CReSA CSFV panels (2022 and 2025) respectively, and the RT-qPCR results provided by the IRTA-CReSA reference laboratory, as well as the Hoffmann et al. 2005 [9], RT-qPCR performed at LSE-LANASEVE in 2025, on the right. Samples H and I of the 2022 panel could be considered positive, negative, or doubtful by the CReSA technical personnel.

Sample B (Margarita strain) from the 2022 interlaboratory panel had a Ct value of 37.07 when initially tested at the IRTA-CReSA reference laboratory under accredited diagnostic conditions. Upon retesting at LSE-LANASEVE using the Hoffmann et al. 2005 [9] RT-qPCR assay, the sample remained positive with a Ct value of approximately 36, supporting the interpretation that the discordant result obtained with the conventional endpoint RT-PCR assay was associated with a low viral load close to the analytical detection limit of the assay.

Sample 3 from the IRTA-CReSA 2025 panel corresponded to a tonsil sample collected from a domestic pig vaccinated with the live attenuated C-strain Classical swine fever vaccine. This sample belongs to a CSFV primary reference panel produced and validated at the IRTA-CReSA laboratory, a WOAH Reference Laboratory for Classical swine fever. The sample had been previously validated using the Hoffmann et al. (2005) RT-qPCR assay under ISO 17025-accredited diagnostic procedures [9], and consistently tested positive in three independent experiments performed in triplicate at the CReSA reference laboratory.

After shipment of the panel to Costa Rica, the samples were held in customs for 3 weeks. After being released the samples were processed at LSE-LANASEVE using the same RT-qPCR strategy but under different amplification conditions, equipment, reagents, and laboratory settings, and yielded a negative result. Considering that the original Ct values of the sample ranged between 30 and 32, the absence of amplification observed during retesting was interpreted as likely associated with low target concentration near the detection limit and interlaboratory analytical variation or ARN degradation, rather than evidence that the original sample was negative.

Therefore, only sample B from the IRTA-CReSA 2022 panel was considered a true discordant result for the diagnostic performance analysis of the conventional pestivirus RT-PCR assay.

### 3.3. Sensitivity and Specificity Diagnostic

Table 1 summarizes the results obtained from the 66 reference samples and the corresponding diagnostic performance estimates.

The diagnostic sensitivity was 98% (95% CI: 89.55–99.95%), and the specificity was 100% (95% CI: 78.2–100.0%). The positive predictive value (PPV) and negative predictive value (NPV) were 100% (95% CI: 92.89–100.0%) and 99.28% (95% CI: 95.19–99.90%), respectively, with an accuracy of 99.47% (95% CI: 93.55–100.00%) for a BVDV-1 prevalence of 27% [18].

### 3.4. Repeatability

**Figure 4:**
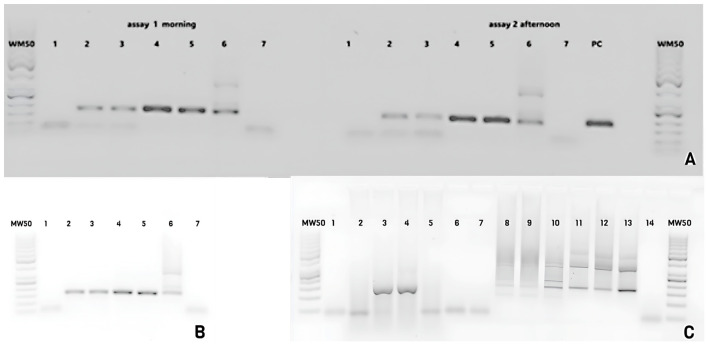
(**A**–**C**) shows the repeatability, reproducibility, and selectivity of the assay.

Figure 4A shows the results of the repeatability assay. MW50 corresponds to the molecular weight marker (50 bp ladder). Lane 1 is the no-template control (water). Lanes 2 and 3 represent the Kanagawa strain M2 (NCFAD 2024 proficiency panel), extracted two years prior. Lanes 4 and 5 correspond to control J (BVDV; IRTA-CReSA 2022 interlaboratory panel). Lane 6 represents a weak positive control. Lane 7 corresponds to sample M13, which is positive for ASFV (Lillie strain; NCFAD 2024 panel). PC indicates a strong positive control.

### 3.5. Reproducibility

Figure 4B presents the results of the reproducibility assay. Lanes 1 to 7 correspond to the same samples used in the repeatability test; however, they were amplified on a different day, using different equipment and a different analyst.

### 3.6. Selectivity

Figure 4C shows the selectivity assay results. Lanes 1 and 14 corresponds to the no-template control, and lane 2 to the negative extraction control. Lane 3 corresponds to tonsil tissue inoculated with the M2 sample (CSFV Kanagawa strain; NCFAD Canada 2024 panel). Lane 4 shows tonsil tissue spiked with sample J (IRTA-CReSA, Spain 2022 panel). Lane 5 corresponds to tonsil tissue spiked with a dengue-positive control. Lane 6 shows negative tonsil tissue, and lane 7 corresponds to sample dengue-positive control 1:10). Lane 8 shows CSFV sample 1:50, lane 9 CSFV sample 1:20, lane 10 CSFV 1:100, lane 11 BVDV 1:50, and lane 12 BVDV 1:20, while lane 13 corresponds to BVDV 1:100 sample.

### 3.7. Pestivirus RT-PCR Surveillance

As a result of routine surveillance conducted from January 2014 to July 2024, a total of 579 samples were tested using the in-house RT-PCR assay. These comprised 11 bovine blood samples and 568 porcine tissue samples (tonsils and lymph nodes). The distribution of samples according to animal species is shown in Table 2.

In porcine, the main reasons for sample submission for pestivirus diagnosis included skin lesions compatible with erysipelas observed at slaughterhouses, deaths during transport, and lymphadenopathies.

Only two of ten pigs, with lesions compatible with erysipelas from a single farm in Monte Verde, Puntarenas, tested positive for pestivirus among the 568 samples analyzed. These samples were collected on 12 June 2015, and were amplified (5′UTR fragment) and confirmed by sequencing on 18 June 2015.

Preliminary identification was performed using BLASTn analysis [15] against GenBank sequences, followed by phylogenetic comparison with representative pestivirus reference strains. Although the analyzed fragment corresponds to a relatively conserved region of the pestivirus genome, the sequences consistently clustered within the BVDV-2a clade and were clearly differentiated from CSFV, BDV, and BVDV-1 reference sequences included in the analysis.

This sequence has a 93.04% of identity with a sequence AF417995 isolated in Argentina. As both Costa Rican sequences were identical, only sequence 10 LSE0797-15 was included in the analysis.

**Figure 5:**
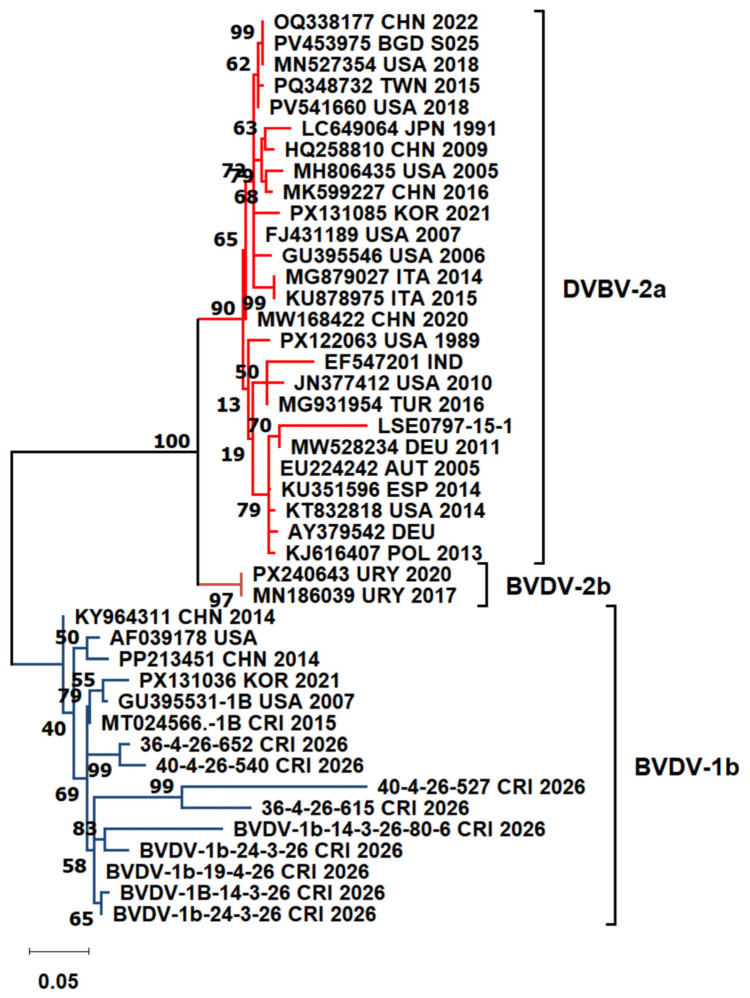
BVDV sequence detected in two pigs in the same farm during the validation of this in-house RT-PCR assay BVDV-2a, as well as nine sequences obtained from bovine isolates in 2026 BVDV-1b.

The nine cell culture supernatants obtained from clinical samples processed at the Virology Laboratory, Veterinary School, Universidad Nacional, tested positive by the pestivirus RT-PCR in house and were subsequently confirmed as BVDV-1b by sequence analysis. Figure 5 shows the phylogenetic tree constructed in MEGA12 using the 10 sequences isolated in Costa Rica together with 33 sequences downloaded from GenBank [17].

The topology of the 43 sequences is divided into two main clusters. One cluster, divided into three groups (red branches), comprises BVDV-2a sequences, including LSE0797-15 from Costa Rica. LSE0797-15 shares a common ancestor with a strain from Germany isolated in 2011 and clusters with sequences from Poland (2013), Spain (2014), Austria (2005), the United States (2014), and another sequence from Germany for which the isolation year is unknown.

The second cluster (blue branches), includes the nine virus isolation sequences, which share a common ancestor with a sequence from Korea isolated in 2021, one from the United States collected in 2007, and another sequence collected in Costa Rica in 2015. All of these sequences were classified as BVDV-1b. The sequences generated in this study have been submitted to GenBank under accession number PZ485860 for the BVDV-2a pig sample and accession numbers PZ502730–PZ502733 and PZ502734–PZ502738 for the nine bovine BVDV-1b sequences.

During the PROCINORTE project, all 493 samples collected from pigs in slaughterhouses between August 2024 and September 9 2025 were negative for pestivirus.

## 4. Discussion

Most molecular assays developed for the detection of pestiviruses or CSFV, including simplex and multiplex systems designed to simultaneously detect ASFV, CSFV, and atypical pestiviruses, are currently based on real-time RT-PCR technology [10,11,12,19,20,21,22]. These assays generally provide excellent analytical sensitivity, with reported detection limits ranging from approximately 2.5 to 10^2^–10^3^ copies/µL depending on the assay format, target region, and detection chemistry employed [10,11,20,21,22,23]. In contrast, conventional endpoint RT-PCR assays, including nested RT-PCR formats, usually show lower analytical sensitivity, with previously reported detection limits around 10^3^ copies for BDV, BVDV-1, and BVDV-2 [21].

In this context, the analytical sensitivity observed for the in-house pestivirus RT-PCR assay developed in this study (1.2 copies/µL for CSFV and 11.7 copies/µL for BVDV) falls within the expected range for endpoint RT-PCR assays and demonstrates adequate performance for surveillance applications. The assay also detected as little as 0.12 TCID_50_ per reaction for the CSFV Honduras strain, which is comparable to previously reported detection limits of 0.32 TCID_50_ obtained with uniplex and multiplex RT-PCR assays [24]. Similarly, a detection limit of 0.89 TCID_50_ has been reported for multiplex assays targeting CSFV and ASFV [23].

Other studies using real-time RT-PCR assays have described detection limits ranging from 0.01 to 3 TCID_50_/mL for CSFV [21,25], whereas higher limits have been reported for BVDV-1 and BVDV-2 detection [13]. Collectively, these comparisons indicate that although endpoint RT-PCR assays remain less sensitive than highly optimized RT-qPCR systems, the assay described here provides satisfactory analytical performance for routine pestivirus surveillance and confirmatory testing. The diagnostic sensitivity and specificity observed in this study were consistent with previously published conventional and real-time PCR assays for pestivirus detection. The only discordant result corresponded to a low viral load sample close to the detection limit, highlighting the expected lower analytical sensitivity of endpoint RT-PCR compared with highly sensitive RT-qPCR assays. In the context of surveillance and confirmatory testing, the high specificity observed in this study is particularly relevant, especially for countries officially free of CSFV, where false-positive results may have important sanitary and economic consequences. According to WOAH recommendations for diagnostic test validation, assays intended for confirmatory diagnosis should prioritize diagnostic specificity while maintaining acceptable diagnostic sensitivity [26].

The repeatability and reproducibility assays yielded consistent results under different analytical conditions, supporting the robustness of the assay for routine laboratory use. In the selectivity assay, an apparent inhibitory effect was observed at high viral concentrations, as suggested by variations in band intensity in Figure 4C. Although this phenomenon did not affect the final interpretation of results, it highlights the importance of matrix composition and viral concentration when interpreting endpoint RT-PCR assays.

An important epidemiological finding of this study was the detection of BVDV in nine bovine isolates classified within the BVDV-1b group and two porcine samples classified within the BVDV-2a group. The nine bovine isolates obtained from clinical cases in 2026 clustered within BVDV-1b, which is consistent with previous reports indicating that BVDV-1b is one of the predominant subtypes associated with respiratory disease in cattle in the Americas [26]. In Costa Rica, previous molecular studies analyzing BVDV isolates collected from dairy cattle between 1987 and 2006 also reported predominance of the BVDV-1b subtype [27]. Therefore, the findings obtained in the present study agree with the historical molecular epidemiology of BVDV reported in the country.

In contrast, the two porcine samples obtained during CSFV surveillance were classified within the BVDV-2a group based on sequence analysis of the amplified 5′UTR fragment and phylogenetic comparison with representative pestivirus reference strains. Although the analyzed fragment corresponds to a relatively conserved genomic region, the sequences consistently clustered within the BVDV-2a clade and were clearly differentiated from CSFV, BDV, and BVDV-1 sequences included in the analysis. Interestingly, the Costa Rican sequence clustered with strains reported in Europe between 2005 and 2014, including sequences from Austria, Poland, and Spain. Notably, the Spanish sequence KU351596 represented the only BVDV-2 strain detected among 86 analyzed BVDV strains in Galicia, whereas the remaining sequences corresponded to BVDV-1 [28]. Similarly, studies conducted within the Austrian national BVDV control program identified only two BVDV-2 sequences among 33 analyzed samples [29].

Although serological evidence of exposure to BVDV-2 had previously been reported in Costa Rica, with a seroprevalence of 19% in cattle sampled in 2008 [18], no confirmed molecular detection of BVDV-2 had been described in the country prior to this study.

BVDV-2 has been associated with outbreaks of acute disease and includes strains with variable virulence ranging from mild to moderate clinical presentations. Although cattle are considered the primary hosts of BVDV, infections in other species, including pigs, have also been reported [30]. In Brazil, a survey conducted in backyard pigs identified two pestivirus-positive samples classified as BVDV-1d and BVDV-2a, respectively [31]. Interestingly, the two BVDV-2a-positive pigs identified in the present study originated from a dairy farming area, and the farm was located near a cheese-processing facility, providing a plausible epidemiological context for interspecies exposure.

Experimental studies have shown that pigs can become infected with both BVDV-1 and BVDV-2, although infections are frequently subclinical. Previous work demonstrated that viremia could be detected in pigs inoculated with BVDV-1, whereas virus isolation was less successful following infection with BVDV-2. These findings suggest that BVDV infections in pigs may occur without evident clinical signs and therefore remain undetected unless specific surveillance activities are conducted [32].

Therefore, the molecular identification of BVDV-2a in pigs represents a relevant epidemiological finding and highlights the importance of broad-range pestivirus surveillance assays capable of differentiating CSFV from other pestiviruses potentially detected in porcine samples [33].

In our laboratory, all samples testing positive by PCR are routinely confirmed by sequencing. This step serves to rule out potential contamination from the positive control or nonspecific amplification products and allows the genetic characterization and lineage assignment of the detected strains [34,35].

The combination of broad-range molecular detection and sequence confirmation makes this approach particularly useful for epidemiological investigations, surveillance programs, and the identification and characterization of emerging viral pathogens [36].

In conclusion, the conventional in-house pestivirus RT-PCR assay demonstrated satisfactory analytical and diagnostic performance for the broad detection of pestiviruses and proved useful for pestivirus surveillance and confirmatory testing when combined with sequence analysis in a CSFV-free country. The study also reports the first molecular detection of BVDV-2a in pigs in Costa Rica, highlighting the epidemiological importance of broad-range molecular surveillance. The detection and characterization of non-CSFV pestiviruses in swine populations can improve differential diagnosis, strengthen early warning systems, and enhance surveillance strategies, thereby supporting more effective CSFV prevention and control programs.

## Figures and Tables

**Table 1:** Results of the 66 processed known samples used to estimate the sensitivity and specificity of the pestivirus assay.

RT-PCR Pestivirus	Reference Samples		
	Positives	Negatives	Total
Positives	50	0	50
Negatives	1	15	16
Total	51	15	66

The McNemar test comparing the results obtained with the in-house pestivirus RT-PCR assay and those from the reference laboratories were not statistically significant (*p* = 1.00). Agreement between methods, assessed using Cohen’s kappa coefficient, was 0.96 (95% CI: 0.88–1.00), with a standard error of 0.04.

**Table 2:** Samples processed for pestivirus from 2014 to 2024.

Species	Samples Tested (n)	Total Population (n)	No. Establishments	Results
Bovine	11	562	5	All negative
Porcine	568	113,427	176	2/568 Positive
Total	579	112,989	181	-

## Data Availability

The original contributions presented in this study are included in the article. Further inquiries can be directed to the corresponding author.

## Supplementary Materials

The following supporting information can be downloaded at: https://www.mdpi.com/article/10.3390/v18070762/s1, Table S1. Reference controls used in this validation.
