# Supplementary material for: Validation of a Broad-Range Conventional RT-PCR Assay for Pestivirus Surveillance and Molecular Detection of BVDV-2a in Pigs in Costa Rica
Source: Viruses. 2026 Jul 11;18(7):762. doi: 10.3390/v18070762 (PMC13431626; doi:10.3390/v18070762)
Supplement: Supplementary file 1 [file viruses-18-00762-s001.zip › viruses-4438741-supplementary.pdf]

Supplementary material

Table S1. Reference controls used in this validation.

**Lab.**		**ID**	**Reference Lab Ct**
Lab NCFAD Winnipeg Canada Internship 2012	1	Kanagawa	25.2
	2	Paderborn	25.4
	3	Peru	21.5
	4	Honduras	32.3
	5	Glentorf	20.8
	6	Alfort	27.3
	7	Parma	26.6
	8	Diepholtz	25.4
	9	Brescia	19.0
	10	Congenital Tremor	31.6
	11	BVDV Singer	17.8
	12	BVDV 125C	18.5
	13	BVDV	16.7
	14	BDV	20.2
	15	Tonsil	Neg
Lab CENSA Cuba 2015	16	1	Neg
	17	2	30.22
	18	3	29.33
	19	4	27.48
	20	5	29.33
	21	6 BVDV	23.9
	22	7	30.22
	23	8	29.33
	24	9	Neg
	25	10	27.48
Lab NCFAD Winnipeg Canada 2024	36	1 ASF Georgia	Neg
	37	2 Kanagawa	25.07
	38	3 Diepholz	25.14
	39	4 Diepholz	28.75
	40	5 T. Medium	Neg
	41	6 Alfort/187	27.28
	42	7 ASF Malawi	Neg
	43	8 Kanagawa	28.65
	44	9 ASF Malawi	Neg
	45	10 Alfort/187	30.85
	46	11 ASF Lilile	Neg
	47	12ASF Georgia	Neg
	48	13 ASF Lilile	Neg
	49	14 T. Medium	Neg
	50	15 Alfort/187	23.89
	51	1 Margarita 1.4	22.5
Lab IRTA CRESA	52	2 Thiverval 1.1	31.75
	53	3 Cstrain 1.1	33.45

Catalunya Spain 2025	54	4 Catalunya 2.3	22.47
	55	5 Alfort/187 1.1	19.5
	56	6 Margarita 1.4	25.93
	57	7 Serum	Neg
Esc Med Veterinaria, UNA Costa Rica 2026	58	BVDV-19-4-26	Positive DIF
	59	BVDV-24-3-26	Positive DIF
	60	BVDV-14-3-26 80.6	Positive DIF
	61	BVDV-14-3-26 289	Positive DIF
	62	BVDV-14-3-26 238	Positive DIF
	63	BVDV-36-4-26 652	Cytopathic Effect
	64	BVDV-36-4-26 615	Cytopathic Effect
	65	BVDV-40-4-26 527	Positive DIF
	66	BVDV-40-4-26 540	Positive DIF
